# Cocaine-induced sensitization and glutamate plasticity in the nucleus accumbens core: effects of sex

**DOI:** 10.1186/s13293-023-00525-8

**Published:** 2023-06-24

**Authors:** Amanda M. Catalfio, Tracy L. Fetterly, Allison M. Nieto, Terry E. Robinson, Carrie R. Ferrario

**Affiliations:** 1 Pharmacology Department, University of Michigan, Ann Arbor, MI USA; 2 Neuroscience Graduate Program, University of California, Berkeley, CA USA; 3 Psychology Department (Biopsychology Area), University of Michigan, Ann Arbor, MI USA

## Abstract

**Background:**

The development and persistence of addiction is mediated in part by drug-induced alterations in nucleus accumbens (NAc) function. AMPA-type glutamate receptors (AMPARs) provide the main source of excitatory drive to the NAc and enhancements in transmission of calcium-permeable AMPARs (CP-AMPARs) mediate increased cue-triggered drug-seeking following prolonged withdrawal. Cocaine treatment regimens that result in psychomotor sensitization enhance subsequent drug-seeking and drug-taking behaviors. Furthermore, cocaine-induced locomotor sensitization followed by 14 days of withdrawal results in an increase in glutamatergic synaptic transmission. However, very few studies have examined cocaine-induced alterations in synaptic transmission of females or potential effects of experimenter-administered cocaine on NAc CP-AMPAR-mediated transmission in either sex.

**Methods:**

Male and female rats were given repeated systemic cocaine injections to induce psychomotor sensitization (15 mg/kg, i.p. 1 injection/day, 8 days). Controls received repeated saline (1 mL/kg, i.p). After 14–16 days of withdrawal brain slices were prepared and whole-cell patch-clamp approaches in the NAc core were used to measure spontaneous excitatory post-synaptic currents (sEPSC), paired pulse ratio, and CP-AMPAR transmission. Additional female rats from this same cohort were also given a challenge injection of cocaine at withdrawal day 14 to assess the expression of sensitization.

**Results:**

Repeated cocaine produced psychomotor sensitization in both sexes. In males this was accompanied by an increase in sEPSC frequency, but not amplitude, and there was no effect on the paired pulse ratio. Males treated with cocaine and saline had similar sensitivity to Naspm. In contrast, in females there were no significant differences between cocaine and saline groups on any measure, despite females showing robust psychomotor sensitization both during the induction and expression phase.

**Conclusions:**

Overall, these data reveal striking sex differences in cocaine-induced NAc glutamate plasticity that accompany the induction of psychomotor sensitization. This suggests that the neural adaptations that contribute to sensitization vary by sex.

## Plain language summary

Females are more vulnerable to substance use disorder than males. However, preclinical studies in females are lacking, particularly in regard to the function of neural regions that mediate reward and motivation such as the nucleus accumbens (NAc). Cocaine-induced changes in excitatory transmission within the NAc play important roles in cocaine-seeking and addiction, but are under-studied in females. Here we found that cocaine treatment enhances NAc excitatory transmission in males, but has no effects on this aspect of NAc function in females. The neural processes underlying addiction may vary according to gonadal sex.

## Highlights

- Females transition to addiction more quickly than males.
- Neuroadaptations in the NAc that accompany psychomotor sensitization underlie behavioral features of drug addiction.
- Cocaine-induced alterations in NAc core glutamate transmission contribute to drug-seeking behaviors, but cocaine-induced plasticity in this region has not been thoroughly examined in females.
- Despite showing robust psychomotor sensitization, cocaine did not alter NAc core glutamate transmission in female rats, but did enhance NAc synaptic transmission in males.

## Background

Sex differences are reported both in the pattern of drug-taking behavior and the development of substance use disorders [1]. For example, in humans, females transition to addiction more rapidly [2, 3] and report stronger withdrawal effects than males [4-6]. Parallel sex differences have been established in rodent models where female rats acquire cocaine self-administration at a faster rate [7, 8], show a greater magnitude of escalation of cocaine intake [9], and display a higher motivation to obtain cocaine after withdrawal than males [10, 11]. These behavioral studies support the idea that the induction and expression of drug-induced alterations in brain function that underlie addiction vary with gonadal sex [12, 13]. Specifically, estradiol enhances both the acute locomotor effects of cocaine [14, 15] and psychomotor sensitization in females [16, 17]. In contrast, male castration enhances locomotor activity after a single injection, but locomotor sensitization requires testosterone [18].

Drug-induced neuroplasticity thought to contribute to the transition to addiction is associated with the development of behavioral sensitization [19, 20]. One manifestation of these alterations is the persistent enhancement in drug-induced psychomotor activity (i.e., psychomotor sensitization) following repeated exposure to addictive substances like cocaine [21]. Also, animals sensitized to psychostimulant drugs more readily acquire psychostimulant self-administration [22-24], show stronger cocaine conditioned place preference [25], and escalate their cocaine intake more quickly than saline pre-treated controls [26]. Thus, psychomotor sensitization is also associated with incentive sensitization. Of particular relevance here, there are sex differences in both the induction of psychomotor [21, 27, 28] and incentive sensitization [10].

Both psychomotor sensitization and enhancements in cocaine-seeking behaviors have been linked to alterations in nucleus accumbens (NAc) glutamate neurotransmission [29-31]. For example, experimenter-administered cocaine treatment regimens that result in psychomotor sensitization enhance excitatory transmission within the NAc shell and core of mice [32, 33] and increase the surface expression of GluA1 and GluA2 AMPAR subunits in the NAc of rats [29, 34]. However, these studies were done exclusively in males. In addition, prolonged withdrawal from long-access cocaine self-administration increases synaptic transmission mediated by calcium-permeable AMPARs (CP-AMPARs) in the NAc core, resulting in enhanced cue-associated drug-seeking [35, 36]. Yet, whether experimenter-administered cocaine treatments that produce psychomotor sensitization result in similar synaptic enhancements in either sex is unknown.

There are sex differences in medium spiny neuron (MSN) anatomy and function within the NAc. Intact females have greater spine density and spine head size in the NAc core then males [37]. This may be due in part to circulating gonadal hormones, as estradiol specifically decreases spine density and spine maturity in females [38, 39]. Interestingly, these sex differences persist when animals are exposed drugs of abuse. Specifically, repeated systemic cocaine exposure increases NAc spine density in both males and females, but the magnitude of this increase is greater in females than males [40]. In addition, there are sex differences in NAc MSN function. Basal excitatory transmission is enhanced in females compared to males and MSN intrinsic excitability varies with the cycle in females, resulting in complex sex differences in MSN firing [41-43]. For example, NAc mEPSC frequency is similar in males and females when recordings are made from females in the diestrus phase of the cycle, but is enhanced in females vs males when recordings are made from females in proestrus or estrus. Together these data suggest that cocaine-induced NAc glutamatergic plasticity may differ in females vs males.

Therefore, in the current study we determined the effects of a sensitizing regimen of cocaine on NAc core glutamatergic synaptic transmission in male and female rats. All measures were made after 14–16 days of withdrawal from cocaine.

## Materials and methods

### Subjects

Male and female outbred Sprague Dawley were purchased from Envigo (Indianapolis, IN) and were 55 days old upon arrival. Rats were allowed to acclimate to the colony room for one week, after which they were handled (once per day, 5 days) prior to start of the first habituation session (details below). Rats were pair housed by sex on a reverse 12-h light/dark cycle (lights off at 0800) and had free access to water and food. The estrous cycle was not monitored. Procedures were approved by The University of Michigan Committee on the Use and Care of Animals in accordance with AAALAC and AVMA guidelines.

### Drugs and reagents

Cocaine HCL was provided by the NIDA drug supply program. All other drugs and reagents were obtained from Sigma (St. Louis MO, USA) or Tocris (Minneapolis MN, USA).

### Cocaine exposure

Rats were assigned to saline (sal) or cocaine (coc) treatment groups, counterbalanced by weight (M sal: *N* = 19; M coc: *N* = 19; F sal: *N* = 24; F coc: *N* = 24). All injections took place in locomotor activity chambers (22.86 cm × 44.45 cm × 28 cm) equipped with infrared beams around the perimeter. Rats were first habituated to the chambers and injection procedures beginning when they were ~ 67 days old. Briefly, 40 min after being placed in the chamber each animal received an injection of saline (0.9%, 1 ml/kg, i.p.) and remained in the chamber for 60 min. This procedure was repeated on two consecutive days. Rats then received 8 consecutive days of either saline or cocaine (15 mg/kg, i.p.) injections, as previously described [29]. Briefly, rats were placed in the locomotor chambers for 40 min, they were then given an injection of saline or cocaine and returned to their home cage 1.5 h later. After the 8th injection rats were left in their home cages undisturbed for 14–16 days. After this, some rats were used for whole-cell patch-clamp recordings while a subset of females were used to examine the expression of psychomotor sensitization in response to a cocaine challenge. Psychomotor activity was assessed by quantifying the total number of beam breaks per 5 min during each session, as an index of locomotion. Time to peak locomotor activity was assessed as the time (per 5 min) it took to reach the largest number of beam breaks.

### Cocaine challenge

Due to the absence of an effect on sEPSC’s in females, despite the development of psychomotor sensitization during the induction phase, we wanted to rule out a possible abatement of sensitization after 14–16 days of withdrawal. Therefore, a subset of female rats pre-treated with cocaine or saline were given a cocaine challenge on WD14-16 (F sal pre-treated *N* = 6; F coc pre-treated *N* = 6). Procedures were based on Oginsky et al. [44]. 14–16 days after the last pre-treatment session, females from cocaine and saline groups were placed back into the locomotor chambers and given increasing doses of cocaine, starting with saline, followed by 7.5 mg/kg and 15 mg/kg cocaine. These injections were given at 40 min, 80 min, and 140 min after animals were placed in the chamber, respectively. Locomotor activity was assessed as the total number of beam breaks per 5 min. Females remained in the locomotor boxes for 1.5 h after the final injection.

### Whole-cell patch-clamp recordings

Established whole-cell patch-clamping approaches were used [45]. Briefly, rats were anesthetized with chloral hydrate (400 mg/kg, i.p.), brains were removed and placed in ice-cold oxygenated (95% O_2_–5% CO_2_) aCSF containing (in mM): 125 NaCl, 25 NaHCO_3_, 12.5 glucose, 1.25 NaH_2_PO_4_, 3.5 KCl, 1 L-ascorbic acid, 0.5 CaCl_2_, 3 MgCl_2_, pH 7.45, 300–305 mOsm. Coronal slices (300 µm) containing the NAc were made on a vibratome (Leica Biosystems VT 1200, Buffalo Grove, IL, USA). Slices were allowed to recover in oxygenated aCSF (30 min, 37 °C), and then maintained at room temperature (30 min) prior to recording. For the recording aCSF, CaCl_2_ was 2.5 mM and MgCl_2_ was 1 mM. All recordings were made from the NAc core and conducted in the presence of the GABA_A_ receptor antagonist, picrotoxin (50 μM). The NAc core was identified using the anterior commissure as a primary landmark (see Fig. 2E cartoon). MSNs were identified by cell body size (~ 15 um in diameter) and by their capacitance (30–60pF) and membrane resistance (30–120 mOhms) after break in. Due to required recording conditions it prevented other measures of membrane properties distinct to MSNs. Spontaneous excitatory post-synaptic currents (sEPSCs) were recorded at a holding potential of − 70 mV (5 min). For all recordings, pipettes were filled with (in mM): 140 CsCl, 10 HEPES, 2 MgCl_2_, 5 Na^+^-ATP, 0.6 Na^+^-GTP, 2 QX-314, pH 7.3, 285 mOsm. Evoked EPSCs (eEPSCs) were elicited by local stimulation (0.02 to 0.30 mA square pulses, 0.1 ms, delivered every 20 s) using a bipolar electrode placed about 300 μm lateral to recorded neurons. The minimum amount of current needed to elicit a synaptic response with less than 20% variability in amplitude was used. If more than 0.30 mA was required, the recording was terminated. eEPSCs were recorded at − 70 mV before and after application of the CP-AMPAR selective antagonist Naspm (200 µM). Paired pulse ratio recordings were recorded at − 70 mV. The inter-stim-interval was 20 ms and the ratio was calculated as the amplitude of the second peak divided by the first. For all data analysis, only cells with an access resistance of less than 30 MΩ were used. Cell parameters (capacitance and membrane resistance) were recorded at the start and end of data collection and only cells with less than 20% change across time were included. Recordings were made at 14–16 days after the last cocaine or saline injection and alternated between slices from males or females and from rats in the saline or cocaine group each day (note that no more than 3 cells were collected from the same rat for a given measure; the number of cells per group are given in the results below).

### Analysis and statistics

Evoked and paired pulse responses were analyzed using Clampfit 10.7 (Molecular Devices). sEPSCs were analyzed using MiniAnalysis (Synaptosoft V.6.0.7; amplitude threshold of 5 pA; decay threshold of < 10 ms) and verified by hand. The minimum detected sEPSC amplitude was set to 5pA. Comparisons were made between data collected within the same cohort of animals (i.e., that received saline or cocaine side by side). *T*-Test, two-way, and three-way ANOVAs using standard general linear models (GLM) or mixed model residual maximum likelihood (REML) followed by Sidak’s and Tukey’s post hoc comparisons were used (Prism 9, GraphPad, San Diego, CA). Interpretation of *p*-values is based on guidelines set forth by the American Statistical Association [46]. Experimenters were not blind to grouping during data acquisition but were during analysis. Ns for electrophysiological measures are reported in the results and based on expected effect size and variance of our primary measures.

## Results

### Repeated systemic cocaine results in locomotor sensitization in male and female rats

**Fig. 1:**
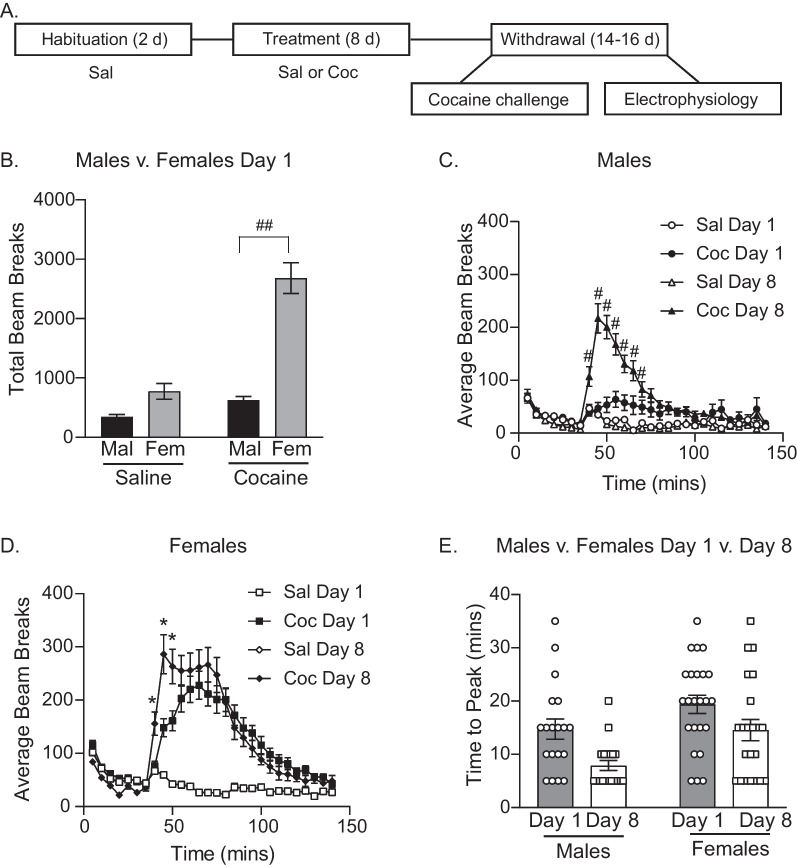
Effects of repeated systemic cocaine on psychomotor behavior in males and females. **A** Schematic displaying the experimental timeline. **B** Comparison of locomotor activity on day 1 between males (Mal) and females (Fem). Females showed a stronger response to the acute locomotor-activating effects of cocaine compared to males. **C** Male and **D** female locomotor behavior on day 1 vs day 8. Animals in cocaine groups (coc) increased locomotor behavior from day 1 to day 8 compared to saline (sal) treated controls. **E** Time to peak locomotor activity on day 1 and day 8 was faster following repeated cocaine treatment. * = Tukey’s post-test *p* ≤ 0.02; ## = Sidak’s post-test *p* < 0.01. All data shown as mean ± SEM

The experimental timeline is shown in Fig. 1A. Females are known to be more sensitive to the acute locomotor-activating effects of cocaine than males [7, 28]. Therefore, we examined total beam breaks in response to the first cocaine injection in females vs males (Fig. 1B). Consistent with previous reports, females showed a stronger acute locomotor response to cocaine than males with significantly greater cocaine-induced locomotion in females than males on day 1 of cocaine exposure (Two-way ANOVA, main effect of sex *F*_(1,82)_ = 57.96, *p* < 0.01; main effect of drug *F*_(1,82)_ = 44.84, *p* < 0.01; drug x sex interaction *F*_(1,82)_ = 24.83, *p* < 0.01: Sidak’s post-test coc treated males vs coc treated females, *p* < 0.01).

Figure 1C, D show beam breaks per 5-min interval after an i.*p*. injection of saline or cocaine, as an index of locomotion, following the first (day 1) and the last (day 8) injection in males and females, respectively (M sal: *N* = 19; M coc: *N* = 19; F sal: *N* = 24; F coc: *N* = 24). As expected, in both sexes cocaine produced a significant increase in locomotor activity compared to animals receiving saline injection (Fig. 1C: three-way REML ANOVA, main effect of treatment *F*_(1,72)_ = 53.62, *p* < 0.01; Fig. 1D: three-way REML ANOVA, main effect of treatment *F*_(91,46)_ = 128.8, *p* < 0.01). The development of locomotor sensitization was assessed by comparing cocaine-induced locomotion in response to the first vs last injection of cocaine within sex. In males, locomotor activity was greater on day 8 than day 1 in rats given repeated cocaine injections, indicative of locomotor sensitization (Fig. 1C: three-way REML ANOVA, main effect of time *F*_(27,1901)_ = 25.58, *p* < 0.01; no effect of day *F*_(1,72)_ = 53.62, *p* = 0.06; time x day x treatment interaction *F*_(27,1901)_ = 10.69, *p* < 0.01; Tukey post-test coc day 1 vs coc day 8 *p* < 0.01 min 40–70). Sensitization was also seen in females, with a greater locomotor response to cocaine on day 8 vs day 1 (Fig. 1D: three-way REML ANOVA, main effect of time *F*_(27,1242)_ = 46.00, *p* < 0.01; no effect of day *F*_(1,46)_ = 0.2497, *p* = 0.62; time x day x treatment interaction *F*_(27,1148)_ = 4.85, *p* < 0.01; Tukey post-test coc day 1 vs coc day 8 *p* ≤ 0.02 min 40–50. However, when comparisons in the magnitude of sensitization were made across sex (i.e., the change in cocaine-induced locomotor activity on day 1 vs day 8), no significant sex differences were found (Fig. 1C, D total beam breaks post cocaine day 1 vs day 8 [95 min]: Two-way REML ANOVA, main effect of day *F*_(1, 80)_ = 4.45, *p* < 0.05; main effect of sex *F*_(1,80)_ = 63.97, *p* < 0.0001; no sex x day interaction *F*_(1, 80)_ = 0.83, *p* = 0.36). This was due in part to the large locomotor response to the first cocaine injection in females, and possibly to the emergence of stereotypy in females by the 8th injection (AMC; see also discussion).

Early studies established that psychomotor sensitization is not only characterized by changes in the magnitude of locomotor activity, but also in the rapidity of onset of psychomotor activity including locomotion [47]. Thus, the time to peak locomotor activity on day 1 vs day 8 was used as a second measure of sensitization in cocaine-treated rats, as this measure may be influenced less by the emergence of stereotyped behaviors (Fig. 1E). Consistent with locomotor results the time to peak was faster on day 8 than day 1 (Fig. 1E: two-way REML ANOVA, main effect of day *F*_(1,39)_ = 11.85, *p* < 0.01). In addition, time to peak was slower in females than males, regardless of day (Fig. 1E: main effect of sex *F*_(1,41)_ = 9.97, *p* < 0.01; no significant sex x day interaction *p* = 0.55). Thus overall, repeated cocaine treatment produced psychomotor sensitization in both sexes, and there was a sex difference in the acute locomotor response on day 1 of cocaine treatment, but no difference in the magnitude of sensitization across sex, at least based on these measures.

### Cocaine exposure and withdrawal did not enhance CP-AMPAR transmission

**Fig. 2:**
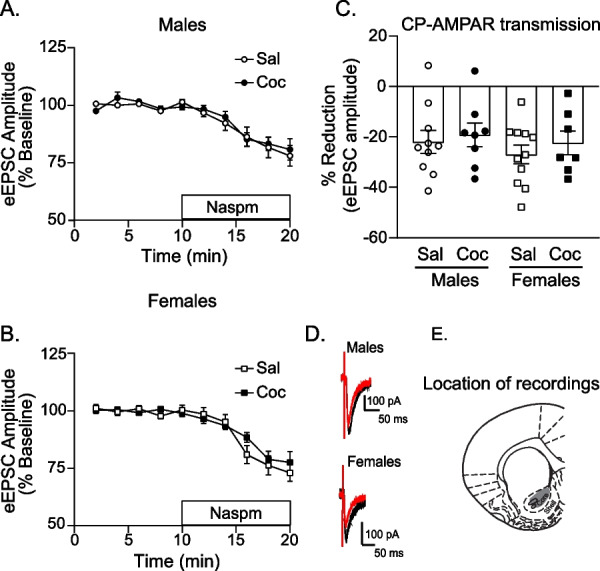
Effects of repeated systemic cocaine and subsequent withdrawal on calcium-permeable AMPAR (CP-AMPAR) mediated transmission. Time course showing effects of bath application of CP-AMPAR antagonist Naspm on eEPSC amplitude recordings in **A** males and **B** females. **C** Percent reduction in eEPSC amplitude following Naspm. Naspm produced similar decrease in eEPSC amplitude in both sexes, regardless of treatment (average of the last two minutes of drug wash on) Two-way ANOVA no effect of treatment *F*_(1,32)_ = 0.68, *p* = 0.42; no effect of sex *F*_(1,32)_ = 0.86, *p* = 0.36. **D** Example traces in males and females. **E** Cartoon depiction of where recordings were made within the slice (shaded region)

Figure 2 shows NAc core CP-AMPAR transmission measured 14–16 days after the discontinuation of cocaine or saline treatments (M sal: *N* = 4 rats, 10 cells; M coc: *N* = 5 rats, 8 cells; F sal: *N* = 5 rats, 11 cells; F coc: *N* = 5 rats, 7 cells). Figure 2A (males) and 2B (females) show the time course of eEPSC amplitude before (baseline; 10 min) and after bath application of the CP-AMPAR antagonist, Naspm (10 min), in saline and cocaine-treated groups. Naspm produced similar decreases in eEPSC amplitude in males and females, regardless of whether they were treated with saline or cocaine (Fig. 2C: two-way ANOVA, no effect of treatment *F*_(1,32)_ = 0.68, *p* = 0.42; no effect of sex *F*_(1,32)_ = 0.86, *p* = 0.36). Example traces before (black) and after (red) Naspm are shown in panel D. Overall, 8 days of cocaine exposure followed by a withdrawal period did not result in changes in CP-AMPAR-mediated transmission in either sex (location of cell recordings is depicted in panel E).

### Cocaine exposure and withdrawal results in sex-specific alterations in sEPSCs

**Fig. 3:**
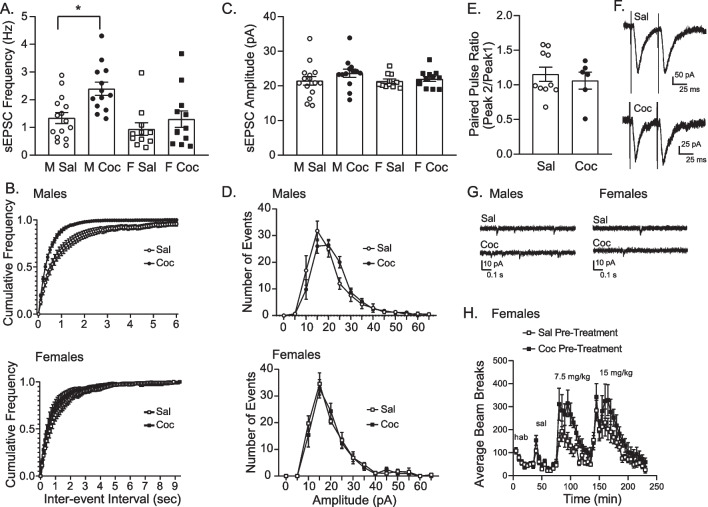
Effects of repeated systemic cocaine and subsequent withdrawal on spontaneous excitatory post-synaptic currents (sEPSCs) and paired pulse ratio. **A** Average sEPSC frequency in cocaine (coc) and saline (sal) pre-treated groups. In males, cocaine treatment increased sEPSC frequency compared to saline treatment. No effects were found in females. **B** Cumulative sEPSC frequency distribution in males (top) and females (bottom). Males treated with cocaine show a clear shift in the sEPSC frequency distribution with more events occurring with shorter inter-event intervals, but no such shift in sEPSC frequency distribution was found in females. **C** Average sEPSC amplitude in cocaine and saline pre-treated groups. sEPSC amplitude was unaffected by cocaine treatment in both groups. **D** sEPSC Amplitude distribution in males (top) and females (bottom). **E** Paired pulse ratio in males. Cocaine treatment in males did alter the paired pulse ratio. **F** Representative paired pulse ratio trace in saline and cocaine-treated males. **G** Representative sEPSC traces in males (top) and females (bottom) from saline- and cocaine-treated groups. **H **Locomotor activity in response to cocaine on withdrawal day 14–16 in saline- or cocaine-pretreated females. Cocaine pre-treated females showed stronger cocaine-induced locomotion at both doses tested compared to saline pre-treated females receiving cocaine for the first time. * = Sidak’s post-test *p* < 0.01; # = main effect of pre-treatment *p* = 0.03

Figure 3 shows sEPSC frequency (A, B) and amplitude (C, D) following withdrawal from repeated cocaine or saline treatment in both sexes (M sal: *N* = 5 rats, 15 cells; M coc: *N* = 4 rats, 13 cells; F sal: *N* = 6 rats, 11 cells; F coc: *N* = 5 rats, 12 cells; representative traces shown in panel G). sEPSC frequency was greater in males than in females regardless of treatment (Fig. 3A: two-way ANOVA, main effect of sex *F*_(1,47)_ = 8.56, *p* < 0.01; no significant drug x sex interaction, *p* = 0.15). In addition, there was a significant main effect of drug (Fig. 3A: *F*_(1,47)_ = 9.66, *p* < 0.01) that was driven by an increase in sEPSC frequency in males treated with cocaine, with no difference between cocaine and saline-treated females (Sidak’s post-test: males sal vs coc, *p* < 0.01; females sal vs coc, *p* = 0.54). In addition, males treated with cocaine show a clear shift in the sEPSC frequency distribution with more events occurring with shorter inter-event intervals (Fig. 3B upper panel: two-way RM ANOVA REML, main effect of drug *F*_(1,25)_ = 6.68, *p* < 0.01), but no such shift in sEPSC frequency distribution was found in females (Fig. 3B lower panel: two-way RM ANOVA REML, no main effect of drug *F*_(1,12)_ = 0.74, *p* = 0.41). Thus, cocaine treatment resulted in an increase in sEPSC frequency in males, but not females.

When average sEPSC amplitudes were examined, no differences were found across treatment groups or sexes (Fig. 3C: no effect of treatment *F*_(1,47)_ = 1.76, *p* = 0.19; no effect of sex *F*_(1,47)_ = 0.68, *p* = 0.41; no interaction *F*_(1,47)_ = 0.53, *p* = 0.47). Similarly, the amplitude distributions remained unchanged in both sexes following cocaine (Fig. 3D: males no effect of treatment *F*_(1,242)_ = 0.01, *p* = 0.92; females no effect of treatment *F*_(1,126)_ = 0.13, *p* = 0.72). Finally, given that there was an increase in sEPSC frequency in males, we next measured the paired pulse ratio to determine if the increase in frequency was associated with an increased probability of pre-synaptic glutamate release (Fig. 3E: M sal: *N* = 3 rats, 10 cells; M coc: *N* = 3 rats, 6 cells; representative traces shown in panel F). However, the paired pulse ratio was similar in males treated with cocaine or saline (unpaired two-tailed *t*-test, *t*_(14)_ = 0.56, *p* = 0.58).

Although many studies have established that psychomotor sensitization persists for many weeks even after a single injection [27, 48], it is possible that the absence of an effect of cocaine treatment on glutamate transmission in females could be due to an abatement of sensitization after withdrawal. To address this possibility, a subset of cocaine and saline-treated females were given a cocaine challenge on withdrawal days 14–16 (Female sal pre-treated *N* = 6; Female coc pre-treated *N* = 6). Cocaine pre-treated females showed stronger cocaine-induced locomotion across both doses tested compare to saline pre-treated females receiving cocaine for the first time (Figure H: two-way RM ANOVA, main effect of pre-treatment, *F*_(1,10)_ = 6.42, *p* = 0.03; main effect of time *F*_(45,450)_ = 15.77, *p* < 0.01; no pre-treatment x time interaction *F*_(45,450)_ = 1.28, *p* = 0.11). This highlights that females pre-treated with cocaine show lasting behavioral sensitization at WD 14–16. Therefore, the absence of effects on NAc excitatory transmission in females is not likely due to an abatement of sensitization.

## Discussion

We examined the effects of a sensitizing regimen of cocaine on NAc core glutamatergic transmission after 14–16 days of withdrawal in male and female rats. There were no effects of cocaine treatment on CP-AMPAR-mediated transmission in either sex. However, males, but not females, treated with cocaine showed an increase in sEPSC frequency. Together these data show that despite the development of robust psychomotor sensitization in both sexes, NAc excitatory transmission was enhanced only in males.

Prior studies established that females show a greater enhancement in locomotor activity both acutely and following repeated cocaine injections compared to male rats [17, 28, 49]. These sex differences in the response to cocaine rely on gonadal hormones. Specifically, estradiol enhances the acute locomotor effects of cocaine [14, 15] and facilitates psychomotor sensitization in females [16, 17]. Furthermore, ovariectomizing females attenuates both the acute [15] and sensitizing effects of cocaine [14]. Here, females were more sensitive to the acute psychomotor-activating effects of cocaine than males (Fig. 1B), consistent with prior studies. Unexpectedly, we did not find evidence for greater psychomotor sensitization in females compared to males. It is unlikely that this is due to procedural differences as the dose and regimen used here is similar to that used in studies where sex differences in the magnitude of sensitization were observed [17, 28]. However, on the 8th day of cocaine injection some females showed brief bouts of in place stereotyped head movements (unpublished observation, AMC). This can interfere with the ability of locomotor-based measures to capture sensitization [50]. Thus, it is possible that the automated beam break measure used here may be an under-estimate of the overall magnitude of psychomotor activity in females [21, 50].

Previous studies in male rats found increases in the surface expression of GluA1 and GluA2 AMPAR subunits in the NAc following cocaine withdrawal [29, 34]. Although these changes in protein expression suggest enhancements in NAc AMPARs, direct measures of AMPAR synaptic transmission were not made. Here we found no effects of cocaine treatment on CP-AMPAR-mediated transmission (Fig. 2) or on sEPSC amplitude in males (Fig. 3C, D). The absence of a change in NAc CP-AMPAR transmission is consistent with results from recordings in the NAc shell of male mice after withdrawal from a sensitizing regimen of cocaine [52]. These data are also consistent with the idea that prolonged withdrawal from long-access cocaine self-administration is required for the synaptic recruitment of these receptors [35, 53], rather than cocaine exposure per se.

The absence of any effect on sEPSC amplitude here is somewhat surprising in light of enhancements in NAc core mEPSC amplitude in male mice [33] and increases in surface protein expression of AMPAR subunits in rat NAc [29, 34]. The former could be due to species differences (mice vs rats here) or other methodological differences (e.g., sagittal vs coronal sections, internal recording solution, and miniature vs spontaneous EPSCs). However, increases in protein expression and null effects on sEPSC amplitude are not mutually exclusive; it is possible for increases in NAc AMPAR subunit protein expression to result in the accumulation of AMPARs at extra-synaptic sites without resulting in increases in synaptic AMPAR transmission [54-56].

The primary effect of cocaine we found in males was an increase in sEPSC frequency (Fig. 3A, B) without a concurrent change in the PPR (Fig. 3E). This pattern is consistent with increases in dendritic spine density, which is expected to result in more synaptic contacts and thus an increase in sEPSC frequency, but does not require a change in release probability at individual synapses [57]. Indeed, it is well-established that passive and self-administered cocaine increases dendritic spine density and excitatory synapse number within the NAc of males and females [58-60]. Furthermore, Wissman et al. also found concurrent increases in NAc dendritic spine density and mEPSC frequency with no changes in PPR following cocaine exposure and withdrawal in males [40]. Thus, data here are consistent with prior results examining effects of experimenter-administered cocaine and subsequent withdrawal on NAc core excitatory synaptic transmission in males.

Surprisingly, despite showing robust sensitization, no effects of cocaine on NAc core glutamatergic transmission were found in females (Figs. 2, 3). One possible explanation for the absence of effects is that sensitization may have abated by the time recordings were made on withdrawal day 14–16. We examined this possibility by re-exposing females pre-treated with cocaine or saline to cocaine on withdrawal day 14–16 and evaluated the expression of locomotor sensitization. Females pre-treated with cocaine showed a stronger locomotor response to cocaine than saline-pretreated controls, confirming that behavioral sensitization persisted through the withdrawal period (Fig. 3H). Therefore, given that the behavioral response is a manifestation of alterations in mesolimbic function [61], the absence of effects on synaptic transmission are not likely due to a loss of sensitization.

To our knowledge, only one previous study similar to the present report has been conducted in females [40]. They reported a greater increase in NAc core mEPSC frequency in female rats compared to male rats treated with cocaine. The dose of cocaine they used was the same as that used here (15 mg/kg), but their treatment regimen was much longer (1 injection/day, 5 days per week for 5 weeks) and the period of withdrawal (17–21 days) was a little longer than here (1 injection/day, 8 days, 14–16 days of withdrawal). This could suggest that females need more exposure to cocaine to drive changes in glutamatergic transmission in the NAc compared to males. Additionally, Wissman et al. reported disruptions in the estrous cycle during the cocaine sensitization regimen, which corresponded to reductions in cocaine-induced locomotor activity. We did not monitor the cycle in our study, but we did not observe any reductions in locomotor activity after repeated injections in females. In addition, 2 injections per day of 15 mg/kg for 5 days are reported to not be sufficient to disrupt the female cycle in rats [16]. Thus, it seems unlikely that the more modest dosing regimen used here (1 injection/day 15 mg/kg for 8 days) produced disruptions in the cycle.

It is possible that some degree of cycle disruption (and presumably of ovarian hormone fluctuations) may be required to observe alterations in mEPSC frequency in the NAc. This idea is consistent with the ability of estradiol to rapidly decrease mEPSC frequency in the NAc core of females but not males [62]. Perhaps naturally occurring fluctuations in estradiol are sufficient to dampen the ability of cocaine to induce increases in sEPSC frequency in females. Conversely, cocaine treatment regimens that disrupt the cycle and concurrent fluctuations in estradiol would be expected to remove this protection. This possibility should be directly tested in future studies. Finally, it should be noted that mEPSCs recorded in Wissman et al. [40] and sEPSCs (recorded here) may be capturing different aspects of synaptic transmission and/or different populations of synaptic inputs. This is mitigated somewhat by the use of coronal sections in our study (which contain glutamate terminals, but lack axon initial segments and cell bodies from glutamate inputs, limiting the likelihood of action potential driven synchronous release), but nonetheless could contribute to the differences observed between these studies.

### Perspectives and significance

Cocaine pre-treatment that produced psychomotor sensitization in both males and females also produced an increase in sEPSC frequency in males, but had no effects on key aspects of glutamate transmission in the NAc core of females. Of course, the NAc core is not the only brain region involved in sensitization and the development of addiction. For example, there could be effects of cocaine on glutamate plasticity in other striatal regions including the NAc shell or dorsal striatum (see [63] for review). Overall, these studies highlight a striking sex difference in the effect of cocaine on one aspect of NAc function. Given the importance of sensitization in the development and persistence of addiction and the lack of systematic studies in females, these data raise important questions about how neural processes underlying addiction may vary according to gonadal sex.

## Data Availability

The datasets during and/or analyzed during the current study are available from the corresponding author on reasonable request.
